# Histological Changes in Ruptured Anterior Cruciate Ligament: A Comparative, Prospective, Observational Study in Different Age Groups and Time of Presentation Since Injury

**DOI:** 10.7759/cureus.68394

**Published:** 2024-09-01

**Authors:** Nabin K Sahu, Bishnu P Patro, Milan Tripathy, Saurav N Nanda

**Affiliations:** 1 Orthopedics, Institute of Medical Sciences and SUM Hospital, Bhubaneswar, IND; 2 Orthopedics, All India Institute of Medical Sciences, Bhubaneswar, Bhubaneswar, IND; 3 Pathology, Kalinga Institute of Medical Sciences, Bhubaneswar, IND; 4 Orthopedics, Kalinga Institute of Medical Sciences, Bhubaneswar, IND

**Keywords:** ligament injury, rupture, repair, histology, anterior cruciate ligament (acl)

## Abstract

Anterior cruciate ligament (ACL) injury is one of the most common sports-related injuries. Because of its intra-capsular location, it has very little chance of healing following injury. The causes of poor healing of ACL tears are mostly due to poor vascularity, disorganized collagen bundles, insufficient myofibroblast proliferation, etc. The healing potential is also varied in different age groups like any other tissue. Here, we studied the histological changes in ACL remnants that occur after ACL injury in different age groups and with varied times of presentation since injury. It was a prospective observational study comparing the histopathology of ACL remnants in 12 subgroups of cases. Healthy synovial lining, presence of inflammatory cells, neo-vascularization, and myofibroblasts are needed for natural healing of ACL. We found a favorable environment for the healing of ACL in younger patients with an injury period of one to three months. We suggest cases with a partial tear of ACL in a young individual presented within one to three months duration may be encouraged for conservative treatment or ACL repair surgery rather than ACL reconstruction. Our initial study on the histopathology of torn ACL has added insight into the existing literature and further studies are needed to substantiate its further application.

## Introduction

The anterior cruciate ligament (ACL) is one of the most important ligaments of the knee joint, providing stability to the knee joint during normal walking and activities like running and jumping. Following injury, every tissue passes through different natural stages of healing. Extra-articular ligaments of the human body heal by a series of inflammatory, proliferative, and remodeling phases resulting in a scar [1]. ACL is a joint ligament prone to injury and strongly associated with sports activity. This intra-articular ligament noted no bridging scar after tearing, resulting in 40-100% nonunion [2,3]. Due to poor healing, ACL tear significantly impacts surgical treatment and post-surgical rehabilitation. Human ACL undergoes four histological phages: (i) inflammatory phase, (ii) epiligamentous reparative phase, (iii) proliferative phase, and (iv) remodeling phase. Angiogenesis and fibroblast proliferation are essential factors for the ACL healing process. A significant difference in the healing of ACL from other ligaments is the formation of alpha-smooth muscle actin, which is on the synovial cell layer on the surface of the ruptured end [3], no tissues for bridging at the torn site, and the presence of epiligamentous reparative phase. Increased transformation rates of collagen synthesis in collagen assays after ACL rupture are independent of the mechanism of injury [4]. Many studies have been noted about the vascularity of ACL, but data about variations in blood vessel density in relation to the duration of injury of ACL are limited [5,6]. Human ACL retracts initially without evidence of healing following ACL injury [7]. Many theories described the causes of poor healing of ACL tears are mostly poor vascularity, disorganized collagen bundles, and insufficient myofibroblast proliferation [8]. It has been noted that surgical repair of ACL has a good outcome in one to two years in most cases [9]; however, there is a high rate of instability, pain, and stiffness in five-year follow-ups [2]. The reason for the non-healing of ACL following injury is still unknown.

The main objective of our study is to compare the histological changes in different age groups at different durations of injury. It can be used to predict the suitable treatment protocol depending upon the age of the patient and time since injury to achieve a good outcome.

## Materials and methods

It was an observational prospective study conducted at a tertiary care hospital after approval from the scientific and ethical committee. Informed consent was obtained from all the patients included in this study.

This study includes all the patients who have undergone arthroscopic ACL reconstruction. A total of 24 patients were included in our study, who were segregated into various groups according to age and duration since injury. Group 1 was less than 30 years old, group 2 was between 30 and 60 years old, and group 3 was more than 60 years old. Again, each group was subdivided into four subgroups. Patients who presented with early injury and were being operated on within one month since injury were included in subgroup A. Similarly, patients being operated on from one to three months, three to six months, and more than six months since injury were subgrouped as B, C, and D, respectively. A total of 12 subgroups with two cases in each subgroup were recruited prospectively. Patients with multi-ligamentous injury, revision surgery, and previous surgery in the knee were excluded from our study. Also, patients with collagen disorder, knee infection, revision knee surgery, deformed knee, and neurological abnormalities were excluded from the study (Table 1).

**Table 1:** Distribution of study groups as per age and duration since injury.

	<30 years of age (1)	30-60 years of age (2)	>60 years of age (3)
<1 month since injury (A)	Subgroup 1A	Subgroup 2A	Subgroup 3A
1-3 months since injury (B)	Subgroup 1B	Subgroup 2B	Subgroup 3B
3-6 months since injury (C)	Subgroup 1C	Subgroup 2C	Subgroup 3C
>6 months since injury (D)	Subgroup 1D	Subgroup 2D	Subgroup 3D

The torn ACL stump was arthroscopically excised from the tibial attachment and was used for histopathological study using hematoxylin & eosin (H&E) stain. Status of cellularity, neovascularization, composition of myofibroblast-like cells, synovial tissue lining at the ruptured site, and collagen content were estimated and observed in all patients. The chief pathologist was the same for all the cases. All histopathological data were evaluated and compared using SPSS (IBM SPSS Statistics for Windows, version 19.0.; IBM Corp., Armonk, NY) software.

All cases were operated by arthroscopic assisted surgery. First, the joint was visualized by arthroscope and the diagnosis was confirmed. Intraoperatively, the torn ACL was taken by arthroscopic basket punch at the site of the tibial attachment. All the specimens were sent to the pathology laboratory, processed, and examined by microscope and immunohistochemistry. By histopathological examination, we got information about cellularity status (synovial lining), neovascularization, inflammatory infiltration, hyalinization, presence of myofibroblasts, and calcification. We have graded these parameters as 0 or 1 as per the absence or presence of the particular parameter in histopathological findings.

## Results

**Figure 1:**
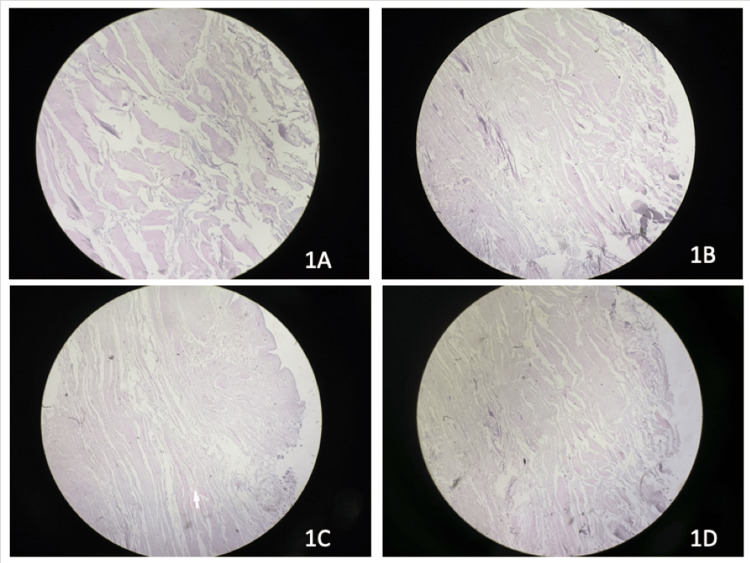
Histological findings of cases with less than 30 years of age (hematoxylin & eosin stain, 100x magnification). 1A: Less than one month since injury. 1B: One to three months since injury. 1C: Three to six months since injury. 1D: More than six months since injury.

In our study, there were 24 patients and 12 subgroups. All the group's results grading parameters are mentioned in Table 2. Group 1A had the presence of synovial lining, neovascularization, and inflammatory cells and the absence of hyalinization, calcification, and myofibroblasts (Figure 1A). Group 1B had flattened synovial lining, neovascularization, myofibroblasts, and hyalinization (Figure 1B). There was no calcification. Group 1C had similar parameters as group 1B (Figure 1C). Group 1D had similar parameters except for the presence of myofibroblast and calcification (Figure 1D).

**Table 2:** Histological findings (based on morphological findings using hematoxylin & eosin stain with 100x magnification).

Group (Case Serial No.)	Synovial lining	Neovascularization	Inflammatory infiltration	Hyalinization	Calcification	Myofibroblast
1A (1, 2)	1, 1	1, 1	1, 1	0, 0	0, 0	0, 0
1B (6, 11)	1, 1	1, 1	1, 1	0, 1	0, 0	0, 1
1C (7, 12)	1, 1	1, 1	1, 1	1, 1	0, 0	1, 1
1D (9, 14)	1, 1	1, 0	1, 1	0, 0	0, 0	0, 0
2A (15, 16)	1, 0	1, 1	1, 1	0, 0	0, 0	0, 0
2B (17, 18)	1, 1	1, 1	0, 0	1, 1	0, 0	1, 1
2C (4, 10)	0, 0	0, 0	0, 0	1, 1	1, 1	1, 1
2D (22, 23)	0, 0	0, 0	0, 0	0, 0	1, 1	0, 0
3A (8, 24)	1, 0	1, 1	1, 1	1, 1	0, 0	0, 0
3B (5, 13)	0, 0	1, 1	0, 0	1, 1	1, 1	1, 1
3C (20, 21)	0, 0	0, 0	0, 0	1, 1	1, 1	1, 1
3D (3, 19)	0, 0	0, 0	0, 0	0, 0	1, 1	0, 0

**Figure 2:**
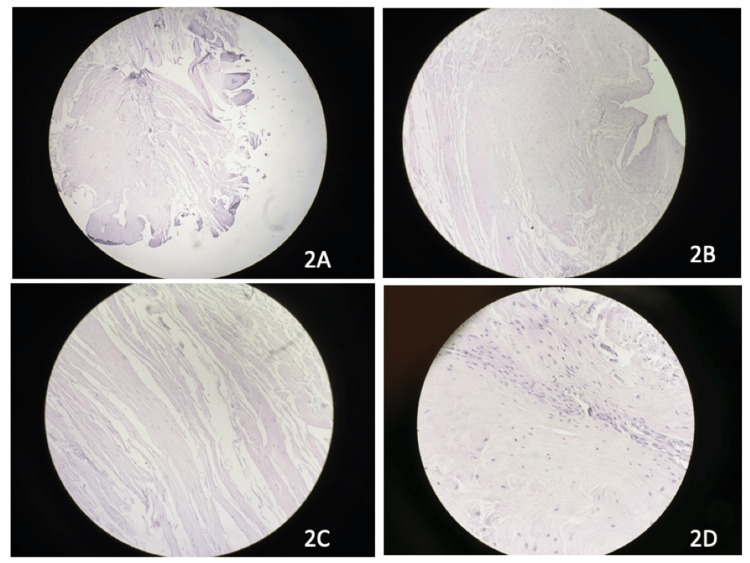
Histological findings of cases with 30-60 years of age (hematoxylin & eosin stain, 100x magnification). 2A: Less than one month since injury. 2B: One to three months since injury. 2C: Three to six months since injury. 2D: More than six months since injury.

Group 2A had flattened synovial lining and fibrotic synovium, mild neovascularization, collagen, and absence of myofibroblast and calcification (Figure 2A). Group 2B had similar parameters as group 1B, except having less inflammatory cells (Figure 2B). Group 2C had no synovial cells, neovascularization, or inflammatory infiltrates with an abundant presence of myofibroblasts and calcification (Figure 2C). Group 2D had an absence of synovial cells, neovascularization, hyalinization, inflammatory infiltrates, and myofibroblasts with an abundance of calcific foci (Figure 2D).

**Figure 3:**
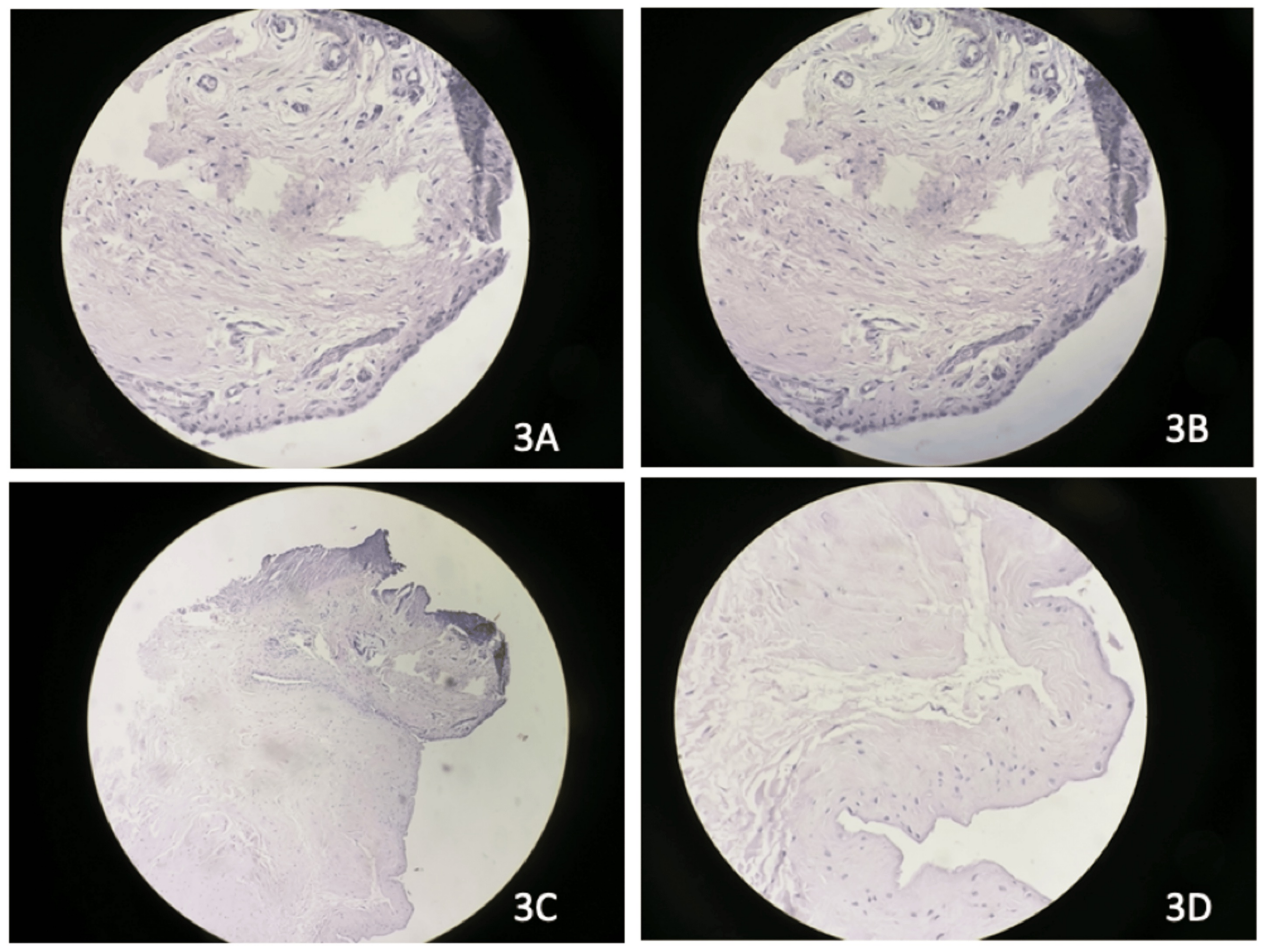
Histological findings of cases with >60 years of age (hematoxylin & eosin stain, 100x magnification). 3A: Less than one month since injury. 3B: One to three months since injury. 3C: Three to six months since injury. 3D: More than six months since injury.

Group 3A had fibrous synovium with mild neovascularization, the presence of inflammatory infiltration and hyalinization with moderately present collagen, and the absence of calcification and myofibroblasts (Figure 3A). Group 3B had no synovium or inflammatory infiltration, with the presence of dense blood vessels, myofibroblasts, and collagen (Figure 3B). Group 3C had similar parameters to group 3B except for the moderate presence of blood vessels (Figure 3C). Group 3D had a presence of abundance of calcification (Figure 3D) (Table 2).

## Discussion

ACL injury is the most common ligament injury of the knee, commonly occurring in young people and persons with sports activity [10,11]. Complete ACL injury has little potential to heal and requires a complete reconstruction of the ACL [12]. In contrast, partial tear of ACL includes a vascularized synovial sheath, plenty of fibroblast and myofibroblast, and mechanoreceptors found in ACL remnants, which gives a knowledge of preservation of remnant ACL in partial reconstruction or augmentation of ACL [13]. The study aimed to compare the histological changes in groups of different ages with different durations of ACL injury. Murray et al. [3] concluded that the human ACL undergoes four histological phases after the torn ACL from the 23 cases of the open reconstruction method: inflammation, reparative regeneration, proliferation, and remodeling. They highlighted some differences in different connective tissue injuries, formation of synovial cell layer on the surface of the torn site, absence of fibrin clot, lack of bridging tissue at rupture site, and presence of epiligamentous reparative phase.

From our study, according to the age and duration of injuries, we briefly got the results indicating the presence of cellularity of the synovium in the younger age group <30 years in less than one month and flattened synovium in one to three, three to six, and six to 12 months. The presence of neovascularization and blood vessels in younger age groups irrespective of the duration of injury was noted, with the presence of hyalinization between zero and six months and absence in six to 12 months of duration of injuries. The presence of myofibroblast was in zero to one month, with a maximum in one to three months and three to six months, and the absence of myofibroblast was in six to 12 months of injury with this younger age group. In the age group of 30-60 years, flattened synovium and fibrous synovium were found within one month and one to three months, whereas the absence of synovium was observed in three to six and six to 12-month durations. There was neovascularization within one month and a maximum in one to three months, whereas there was an absence of neovascularization in three to six and six to 12 months. Collagen fibrils were densely present in one and one to three months, whereas collagen fibrils were visible in three to six and six to 12 months. Blood vessels were found in all stages of duration. Myofibroblast was absent in one month and six to 12 months while maximum in three to six and six to 12 months of duration. In the age group above 60 years, there was fibrous synovium within one month and no synovium in other groups. We found neovascularization during one to three months and absence in the rest of the groups.

Nayak et al. observed fibroblast density to be least in group I, which was less than six weeks coinciding with the epiligament reparative phase, significantly increased in seven to 12 weeks, signifying the proliferative phase, reached a maximum at 13-20 weeks, then reduced after 20 weeks signifying remodeling and maturation phase [10]. They found that blood vessel density was maximum at 13-20 weeks, which coincides with the proliferative phase, and minimum after 50 weeks, which coincides with the reorganization phase when the pro-angiogenic factors were reduced. They also found a decrease in collagen between seven and 12 weeks, then increased after 12 weeks [10]. They proposed that 13-20 weeks of ACL injury would be the ideal time for ACL reconstruction with remnant ACL as favorable fibroblastic and vascular proliferation seen in this phase.

Murray et al. found dilated arterioles and venules with hyperplasia of smooth muscle cells during the phases of ACL healing [3]. They found decreased inflammatory cells but increased fibroblasts at three weeks, whereas at eight weeks, an increase in cell numbers in which fibroblasts are highest among all cell types and maximum cell density was seen at 16-20 weeks. They observed the shape of fibroblasts was fusiform and aligned along the axis of the ligament. In the medial collateral ligament of rabbits, it was found that fibroblast proliferation occurs in the inflammatory phase, whereas the epiligamentous repair phase in ACL failed to heal. Contractile myofibroblasts were found based on anti-alpha smooth muscle actin antibody reactive cells in the synovium during eight to 12 weeks of the repair phase. They also found collagen fascicles were well aligned along the axis of the ligament at around one to two years [3].

Everhart et al. found that at four weeks of duration of acute tear and chronic tear of four years of ACL, cell density increased, which contradicted the theory of hostile synovium. They studied collagen crosslink and found high collagen turnover in both acute and chronic injuries irrespective of duration of injury, mode of injury, and age of the patient. It demonstrated that healing did not stop at a continuous remodeling phase that was going on at any duration during the injury. A higher probability of collagen crosslink was noted if operated for less than 12 months. Still, histology showed a high turnover of collagen fibrils at four weeks duration of injury, which contradicted the theory of hostile synovium [4].

Trocan et al. assessed the blood vessel density of the torn ACL and compared it with intact ligaments using CD34. They found that CD34 positivity was seen in small synovial vessels with high intensity in the endothelium and perivascular cells of the synovium with granular and discontinuous patterns with moderate intensity. Fusiform cells are in the periphery of the ligament close to the synovium, and the fibrocartilage shows a discontinuous pattern with moderate intensity. There were significant differences between the two groups, one with microlesions and the other without microlesions, in which the mean blood vessel density in ligaments with microlesions was found to be 43 per field at x200, compared to 15 per field at x200 in ligaments without injury. In synovium, newly formed vessels were found near the epithelium of the epiligament and were mostly surrounded by CD34-positive fibroblasts. Active neovascularization was determined as marked differences were seen in the size, shape, and thickness of the wall of the blood vessels [14].

Georgiev et al. studied in detail the anatomy of the proximal and distal portion of ACL and stressed on the epiligament theory of healing. They concluded that the epiligament contains more number of cells than the ligament proper and the cell count is more denser in the proximal and distal third than in the central region. The expression of CD34 was more pronounced in the distal third, whereas alpha-smooth muscle actin was more pronounced in the proximal third [15].

The study has some limitations. The sample size of the study population is small. It is an observational design with no intention of randomization. The follow-up period is also brief. We recommend additional research to address all of these limitations. A randomized trial with a bigger sample size and longer follow-up is required to confirm the findings.

## Conclusions

In our study of histological changes of ACL at different durations of injury and different age groups, we concluded that torn ACL in the younger age group of <30 years have better histological healing potential as compared to the other groups due to the presence of neovascularization, cellularity, presence of blood vessels, presence of collagen, and myofibroblasts. The favorable healing environment is maximum at one to three months of duration of ACL injury from our study. We suggest cases with a partial tear of ACL in a young individual presented within one to three months of duration may be encouraged for conservative treatment or ACL repair surgery, rather than ACL reconstruction. This will decrease the burden of operative intervention with a predictable outcome. Our initial study on histopathology of torn ACL has added insight into the existing literature, further studies are needed to substantiate its further application.
